# Comment on Chwal et al. On-Target Low-Density Lipoprotein Cholesterol in Adults with Diabetes Not at High Cardiovascular Disease Risk Predicts Greater Mortality, Independent of Early Deaths or Frailty. *J. Clin. Med.* 2024, *13*, 7667

**DOI:** 10.3390/jcm14082559

**Published:** 2025-04-08

**Authors:** Folkert H. van Bruggen, Hendrika J. Luijendijk

**Affiliations:** Department of Primary and Long-Term Care, University Medical Centre Groningen, University of Groningen, P.O. Box 196, 9700 AD Groningen, The Netherlands

The ELSA-Brasil study provides important insights into the association between LDL-C levels and all-cause mortality in adults with diabetes who are not at high cardiovascular risk [1]. The study reported a U-shaped curve, with the lowest mortality risk at an LDL-C level of 112.2 mg/dL (2.9 mmol/L). Patients with LDL-C levels < 70 mg/dL (1.8 mmol/L) had a significantly higher mortality risk compared to the reference group (100–129 mg/dL, 2.6–3.3 mmol/L) (adjusted hazard ratio [HR] 2.27; 95% CI: 1.51–3.41). The mortality risk was even greater for those with LDL-C < 55 mg/dL (1.4 mmol/L) (adjusted HR 2.69; 95% CI: 1.17–6.17) [1]. In the detailed discussion, the authors cite several observational cohort studies with similar findings.

Additionally, we would like to highlight the results of the prespecified analysis of diabetic patients in the FOURIER trial [2]. It provided valuable information about the risk of all-cause mortality in 11,031 diabetic persons with (very) high cardiovascular risk profiles at very low LDL-C levels. The FOURIER trial evaluated evolocumab versus placebo in patients with established cardiovascular disease who were already receiving statins, ezetimibe, or both. The subgroup of diabetic patients in the evolocumab group, which achieved an average LDL-C level of 31 mg/dL (0.8 mmol/L), exhibited a 10% increase in all-cause mortality risk (HR 1.10; 95% CI 0.91–1.32).

Despite the lack of statistical significance, this observed effect remains noteworthy. First, it contrasts with the effect on the risk of major cardiovascular events (the primary outcome) in diabetic patients, which showed a significant 17% reduction (HR 0.83; 95% CI 0.75–0.93) [2]. It is remarkable that this substantial reduction did not translate into a lower risk of all-cause mortality but instead appeared to coincide with an opposing effect on all-cause mortality.

Second, the increase in all-cause mortality risk was not observed in 16,533 patients without diabetes who achieved the same average LDL-C level (HR 0.99; 95% CI 0.82–1.20) [2]. Therefore, the observed increase in the risk of all-cause mortality in the evolocumab group, starting from 2 years of follow-up, appears to be primarily driven by deaths among diabetic patients. This trend might have reached statistical significance with a longer follow-up, but the early termination of the trial prevented its full assessment [3].

Combined with the findings from the ELSA-Brasil study, these results suggest that very low LDL-C levels may increase all-cause mortality risk in patients with diabetes, regardless of their cardiovascular risk. The ODYSSEY OUTCOMES trial could have provided relevant evidence as well. However, in contrast to statistical analysis plan outlined in the study protocol, the prespecified analysis focusing on patients with diabetes did not report the effect on all-cause mortality [4]. A potential mechanism contributing to the increased mortality risk among diabetic patients may be a heightened vulnerability to infection-related mortality, which has been shown in observational studies [5,6]. The observed increased mortality risk underscores the need for more research to further uncover the mechanisms driving this phenomenon and broadens the call for a cautious approach to setting very low LDL-C targets for diabetic patients [1].
